#  Correlates of Caregiver Burden among Family Members of Patients with Schizophrenia in Lagos, Nigeria

**DOI:** 10.1155/2013/353809

**Published:** 2013-10-03

**Authors:** Increase Ibukun Adeosun

**Affiliations:** Federal Neuro-Psychiatric Hospital Yaba, 8 Harvey Road, PMB 2008, Lagos, Nigeria

## Abstract

Family members of patients with schizophrenia have enormous roles in the care of their patients, which could negatively impact their well being. Development of interventions targeted at alleviating the burden of informal care giving is hinged on the recognition of the factors associated with the various dimensions of burden. This study determined the correlates of caregiver burden among family members of patients with schizophrenia in Lagos, Nigeria. The study instruments included the Zarit burden interview (ZBI) and the positive and negative syndrome scale for schizophrenia (PANSS). Exploratory factor analysis of the ZBI produced a five-factor structure with “financial/physical strain”, “time/dependence strain”, “emotional strain”, “uncertainty”, and “self-criticism” domains. On multiple regression analyses, total PANSS scores, poor social support, and lower educational levels of caregivers were predictive of higher burden scores on the “financial/physical strain”, “time/dependence”, and “emotional strain” domains. Longer duration of illness, shorter patient-caregiver contact time, and being a female caregiver were predictive of higher burden scores on the “uncertainty”, “self-criticism”, and “emotional strain” domains, respectively. There is need for interventions to alleviate the burden on caregivers of patients with schizophrenia in Nigeria. These strategies must include comprehensive social support and improve access to services for patients and their caregivers.

## 1. Introduction

Schizophrenia is a severe mental illness estimated to affect 1 out of 100 people globally [1]. It is a leading contributor to the global burden of disease accounting for about 1% of disability-adjusted life year, 3% of year lived with disability and is the 8th leading cause of disability in people aged 15 to 44 years [2]. The impairment caused by schizophrenia limits the ability of the sufferers to remain independent in various domains of psychosocial functioning. Patients with schizophrenia, therefore, require long-term support and care which may become burdensome to their caregivers.

In many resource-poor countries, community-based mental health services and effective formal support system are unavailable to cater for the needs of patients with schizophrenia. Therefore, the trend towards shorter hospital stay and reduction of in-patient beds have shifted the responsibility of the day-to-day care of patients with schizophrenia from formal caregivers in mental health institutions to informal caregivers within the family setting. The tasks involved in rendering care to a family member with schizophrenia are enormous, and caregivers may become overwhelmed by the demands associated with these roles.

The burden of caregiving is a complex multifaceted construct which may defy a uniformly agreed simple definition [3]. Caregiver burden refers to a psychological state that ensues from the combination of physical work and emotional and social pressure involved in caring [4]. It has also been described as the emotional, social, financial, or physical investment and psychological experiences in reaction to the changes and demands that result from rendering help and support to another person who is not capable of caring for himself or herself by reason of infirmity or disability [3–5]. The psychological consequences of caregiving including emotional reaction, personal appraisal of caregiving experiences, and perceived severity constitute subjective burden. On the other hand, the outwardly quantifiable demands placed on the caregiver including tasks and resources foregone in the course of rendering care are referred to as objective burden [6, 7].

Previous researches have shown that informal caregivers of patients with schizophrenia in Europe, America [8], Australia, [9, 10] Asia [11], and Africa [12, 13] experience moderate to high levels of caregiver burden. However, the socio-demographic and clinical factors associated with the burden of caregiving vary across studies. The factors that tend to be consistently associated with higher levels of burden across studies include the severity of symptoms or psychopathology in the patient [3, 11], lower educational attainment in the caregivers [11, 14], and poor social support [5, 12].

There is limited information on the correlates of caregiver burden on family members of patients with schizophrenia in Nigeria [12, 15–18]. Studies conducted in the western world may have limited generalisation to a low-income sub-Saharan African setting due to sociocultural differences and the disparities in formal support services available to patients with schizophrenia and their caregivers. Furthermore, despite the evidence that caregiver burden is a multidimensional construct, the majority of the previous studies evaluated the correlates of caregiver burden using single global burden rating scores. The impact of the different aspects of burden may vary among caregivers with similar total burden scores. Therefore, there is need to assess the factors associated with the various dimensions of caregiver burden. 

The current study determined the characteristics of caregivers and patients associated with the various dimensions of burden among caregivers of patients with schizophrenia in Lagos, Nigeria. The study hypothesis is that the extent of caregiver burden is determined by the characteristics of the patients and their caregivers and the level of available social support.

## 2. Materials and Methods

### 2.1. Study Location

The study was conducted at the out-patient clinic of the Federal Neuro-Psychiatric Hospital, Yaba, Lagos, Nigeria. The Hospital is the largest psychiatric care facility in the country with weekly out-patient clinic attendance of about 1,000 patients. Though majority of patients are from Lagos and neighbouring states in the Southwest, the hospital has no defined catchment area.

### 2.2. Subjects

Data were obtained primarily from caregivers (*n* = 181) recruited while accompanying the patient to the clinic. However, patients (*n* = 181) were also interviewed alongside their caregivers in order to obtain their clinical characteristics. Only one caregiver was recruited per patient. Inclusion criteria for the caregivers included being resident with the patient, involvement in the care of the patient for at least one year, and age above 18 years. Caregivers with history of preexisting psychiatric illness or caring for other persons with chronic physical or psychiatric illness apart from the patient with schizophrenia were excluded. The patient being cared for must have been diagnosed with schizophrenia for at least a year and have no history of comorbid chronic medical or neurological illness.

### 2.3. Procedure

Approval for the study was obtained from the Research and Ethical Committee of the Federal Neuro-Psychiatric Hospital, Yaba. Patients with schizophrenia accompanied to the clinic by their caregivers were consecutively recruited to participate in the study, subject to their informed consent. Altogether, 188 patients-caregivers dyads were approached, but seven of them declined participation. The diagnoses of the patients were ascertained with the mini international neuropsychiatric interview (MINI) [19]. Caregivers completed the Zarit burden interview [20] and a sociodemographic questionnaire while the patients were assessed by the researcher using the positive and negative syndrome scale for schizophrenia (PANSS) [21], an interviewer-based instrument. Information on some other clinical characteristics of the patients such as age at onset of illness, number of episodes, and number of hospitalisations were supplemented from the case notes. Participants who were illiterate were assisted to complete the questionnaires by reading out the questions and response options to them. 

### 2.4. Measures

#### 2.4.1. Sociodemographic Questionnaire

This was designed by the author to elicit data regarding the sociodemographic characteristics of the participants such as age, gender, household composition, highest level of formal education, occupation, employment status, relationship of the caregiver to the patient, the estimated number of hours of contact with the patient per day, and the perceived level of social support.

#### 2.4.2. Positive and Negative Syndrome Scale (PANSS) [21]

This was used to assess certain clinical characteristics in the patients with schizophrenia. It includes a structured interview to assess patients on 30 items covering positive and negative symptoms as well as general psychopathology. Of the thirty items included in the PANSS, seven constitute a positive scale, seven a negative scale, and the remaining sixteen a general psychopathology scale. For each item, ratings are made on a 1–7 scale of increasing levels of psychopathology ranging from absent to extreme. The scores for the scales are arrived at by summation of ratings for the component items. Therefore, the potential ranges for the positive and negative scales are 7–49 and 16–112 for the general psychopathology scale. The instrument has been used by several authors in Nigeria.

#### 2.4.3. Zarit Burden Interview (ZBI)

This is a 22-item instrument that includes the items most frequently mentioned by caregivers as problem areas in providing care for patients with chronic mental illness (http://www.healthcare.uiowa.edu/igec/tools/caregivers/burdenInterview.pdf). The ZBI explores the negative physical, mental, social, and economic impacts of caregiving on the life of the caregiver. The responses are rated on a Likert scale of 0 (never) to 4 (almost always) with a total score of 0–88. Higher scores indicate higher levels of caregiver burden or distress. Though the ZBI was initially developed to assess caregiver burden in dementia, it has also shown satisfactory psychometric properties in assessing caregiver burden in schizophrenia. The instrument has been widely used to assess caregiver burden on family members of patients with schizophrenia in Asia [22], South America [23], and Africa [13, 15–18]. The popularity of the ZBI in these settings has been attributed to its ability to characterise the sociocultural dynamics of the population to which it is applied and the clarity of the items [23].

#### 2.4.4. Mini International Neuropsychiatric Interview (MINI), English Version 5.0.0 [19]

This was used to ascertain the diagnosis of schizophrenia in the patients. The MINI was designed as a brief structured interview for the major axis 1 diagnosis in the DSM-IV and ICD 10. Validation and reliability studies done comparing the MINI to other similar structured interviews such as the structured clinical interview for the DSM-IV patient version (SCID-P) and the composite international diagnostic interview [24] have shown high validity and reliability scores.

### 2.5. Statistical Analysis

IBM-SPSS software, version 20, was used for the statistical analysis. The dependent variable was the level of burden on caregivers of patients with schizophrenia. A principal component exploratory analysis with varimax rotation was conducted on the items of the ZBI. In order to determine the correlates of the various dimensions of caregiver burden, each of the components/domains derived on factor analysis were used as dependent variables. The independent variables included the sociodemographic characteristics of the caregivers and the patients and the clinical characteristics of the patients. On univariate analysis, the mean values of each of the burden domain scores were compared across dichotomised groups (employed versus unemployed, female versus male, educational attainment below secondary school versus secondary level of education and above, caregivers who were parents or spouses of the patients versus other categories of caregivers, and poor versus good social support) using independent *t*-test. Pearson's correlation analysis determined the association between the burden domain scores and the continuous independent variables. Parametric statistics were used because the burden domain scores were fairly normally distributed. The independent variables were entered into a step-wise multiple regression analysis in the following order: (1) sociodemographic characteristics of the caregivers and patients and (2) clinical characteristics of the patients. Multicollinearity was assessed in the regression analysis with the values of the variance inflation factor and “tolerance”. The entire test was 2-tailed, and the level of significance was set at *P* < 0.05. 

## 3. Results

The sociodemographic characteristics of the patients and caregivers are shown in Table 1. The caregivers had a mean age of 44.8 (±8.3) years and were predominantly females. The patients had a mean age of 39.2 (±9.2) years. The majority (70.2%) of them were unemployed. The level of support available to the patient with schizophrenia was rated as poor by the majority (64.6%) of the respondents. The mean scores of the patients on the positive symptoms scale, negative symptom scales scores, and general psychopathology scales of PANSS were 21.87 (±6.5), 13.33 (±3.2), and 35.63 (±9.4), respectively (Table 2). 

Exploratory factor analysis of the ZBI produced a five-factor structure (Table 6). Based on the items that loaded heavily on these factors, they were termed “financial/physical strain” (items 10, 11, 12, 15, and 17), “time/dependence strain” (items 1, 2, 4, and 6), “emotional strain” (items 3 and 9), “uncertainty” (items 7 and 19), and “self-criticism” (items 21, 22) domains/components. Internal consistency (Cronbach's alpha) of the components were *α* = 0.814 “financial/physical strain”, *α* = 0.751 “time/dependence strain”, *α* = 0.913 “emotional strain”, *α* = 0.584 “uncertainty”, and *α* = 0.849 “self-criticism”. The mean burden scores on each of these domains are shown in Table 3.

Female caregivers had significantly higher burden scores than male caregivers on the “financial/physical strain” (*t* = 2.18, df = 179, *P* = 0.036), “emotional strain” (*t* = 4.63, *P* < 0.001), and “uncertainty” (*t* = 2.97, *P* = 0.024) domains. Family size (number of people living in households) correlated negatively with higher levels of caregiver burden on the “financial/physical strain” (*r* = −0.167, *P* = 0.025) and “time/dependence” (*r* = −0.174, *P* = 0.019) domains (Table 4). The number of hours of contact of the caregivers with the patient inversely correlated with the burden scores on the “self-criticism” domain (*r* = −0.547, *P* < 0.001).

Unemployed caregivers had higher burden scores on the “financial/physical strain” (*t* = 3.04, *P* < 0.003) domain, while employed caregivers scored significantly higher on “time/dependence” (*t* = −4.32, *P* < 0.001) and “self-criticism” domains (*P* = 0.041). Caregivers with lower educational attainment (less than secondary school) had higher burden scores on the “financial/physical strain” (*t* = 4.61, df = 179, *P* < 0.001), “emotional strain” (*t* = 5.08, *P* < 0.001), and “time/dependence” (*t* = 4.09, *P* < 0.001) domains.

Poor social support was associated with higher burden scores on the “financial/physical strain” (*t* = 4.89, df = 179, *P* < 0.001), “emotional strain” (*t* = 5.74, *P* < 0.001), and “time/dependence” (*t* = 5.33, df = 179, *P* < 0.001) domains. Older caregivers had higher burden scores on the “uncertainty” (*r* = 0.304, *P* = 0.002) domain. Caregivers who were parents or spouses had significantly higher scores on the “uncertainty domain” than other caregivers (*t* = 2.91, df = 179, *P* = 0.04). Total PANSS scores correlated positively with higher levels of burden scores on all the domains (*P* < 0.001) except the “uncertainty” domain. Longer duration of illness correlated with higher burden scores on the “financial/physical strain” (*P* = 0.008), “time/dependence” (*P* = 0.011), and “uncertainty” (*P* < 0.001) domains.

In the final regression model, total PANSS scores, poor social support, and lower educational levels of caregivers were all predictive of higher burden scores on the “financial/physical strain”, “time/dependence”, and “emotional strain” domains (Table 5). Longer duration of illness, shorter contact time of the caregiver with the patient, and being a female caregiver were predictive of higher burden scores on the “uncertainty”, “self-criticism”, and “emotional strain” domains, respectively.

## 4. Discussion

The current study assessed the correlates of caregiver burden on family members of patients with schizophrenia, across five domains based on the factor structure of the ZBI in the current sample. Studies on the factors associated with caregiver burden among patients with schizophrenia in Africa are sparse; rare still are studies evaluating the determinants of the various domains of burden on caregivers.

 Female caregivers had significantly higher burden scores on the “emotional strain” and “financial/physical strain” domains. This probably reflects the sociocultural expectations that are placed on females to adopt the caring role whenever a family member becomes ill, regardless of the difficulties in combining the demands of care giving with other enormous socioculturally designated domestic responsibilities. In a patriarchal African society, female caregivers may tend to accept the caregiver role as their exclusive preserve such that the demands on their time by the tasks of caregiving are perceived as normal, thus, accounting for the lack of association of higher burden scores in the “time/dependence” domain with being a female caregiver. 

Caregivers of patients living in households with fewer numbers of people had higher burden scores on the “financial/physical strain” and “time/dependence” domains. This finding highlights the protective nature of multigenerational family system in Africa, wherein the task of caring for an ailing family member is shared by larger number of people beyond the typical nuclear family [12, 25]. Lower educational attainment by the caregiver was predictive of higher burden scores in various domains. This is consistent with previous research [11, 14]. Higher educational attainment confers some degree of socioeconomic advantage and facilitates exposure to wider social network and resources that may mitigate the negative impacts of caregiving. 

Previous authors have noted that the impact of longer caregiver-patient contact time on the burden of care may be attenuated by a larger family size [11]. Consequently, contrary to findings in the western world, longer hours of caregiver-patient contact may not correlate with higher levels of caregiver burden in developing countries with typically large households [9, 12, 25]. In keeping with previous research in nonwestern populations, the findings of the current study suggest that longer duration of patient-caregiver contact is not predictive of higher levels of caregiver burden [12, 25]. On the other hand, shorter caregiver-patient contact time was independently associated with higher burden scores on the “self-criticism” domain. Due to the sociocultural sense of obligation to care for sick family members oneself (rather than delegating care), caregivers who spend lesser time with the patient may be vulnerable to self-reproach.

 Employed caregivers had significantly higher burden scores on the “self-criticism” and “time/dependence” domains. This probably reflects the strain involved in striking a balance between fulfilling the sociocultural expectations of caregiving and the need to go to work in order to earn a living. On the other hand, unemployed caregivers reported higher levels of burden on the “financial/physical strain” domain. Unemployed caregivers are more likely to spend more time within the household and are usually saddled with more caregiving tasks, in comparison to employed caregivers who have longer periods of respite while away at work. Furthermore, the financial obligations associated with caregiving will be more burdensome to a caregiver without a regular source of income [26]. This is particularly important in a low-resourced setting like Nigeria where comprehensive health insurance scheme is lacking and mental health care can only be procured by “out of pocket payment” [27]. 

Poor social support was predictive of higher burden scores on the “financial/physical strain”, “emotional strain”, and “time/dependence” domains. This is consistent with previous reports globally, though the consequences are likely to be more farreaching in settings like Nigeria where formal social support and welfare services for patients with schizophrenia are nonexistent [5, 12, 28, 29]. Community mental health services such as supervised housing, sheltered accommodation, day care services, and domiciliary care are also lacking. Therefore, the day-to-day care of the patients rests completely on available family members. 

Older caregivers and caregivers who were parents or spouses of the patients had significantly higher burden scores on the “uncertainty” domain. Longer duration of illness also correlated with higher scores on the “uncertainty” domain and was the only predictor variable on regression analysis. As caregivers come to terms with the chronic nature of schizophrenia and the reality that their caregiving role may last a lifetime, they may become more worried about the future. Older caregivers may be particularly concerned about who will step into their caregiving roles when they are no longer alive, while spouses may feel entrapped or ambivalent about their choice to remain with the patient. 

Higher PANSS scores predicted higher caregiver burden scores in several domains. The significant association between caregiver burden and the presence of psychopathology is consistent with previous studies [3, 11, 15, 30, 31]. Patients with worse symptom profile may have greater impairment in functioning, thereby, eventuating in the transfer of a greater degree of responsibility to their caregivers. The correlation between symptom severity and caregiver burden underscores the need to ensure effective treatment for patients with schizophrenia as a vital step in addressing caregiver burden. Policy makers need to pay attention to improving access of service users to mental health services.

The cross-sectional design of the study limits assertions on causal relationships between the independent variables and the burden of caregiving. Only available caregivers that accompanied patients to the hospital were studied; this may limit generalisation of findings to the general population. The study also lacked a control group. The strength of the study includes its moderate sample size, assessment of various dimensions of caregiver burden, and the utility of standardised instruments in the ascertainment of the diagnosis and symptoms of schizophrenia in the patients. 

## 5. Conclusion

Diverse characteristics of patients with schizophrenia and their caregivers are associated with various dimensions of caregiver burden. The most recurrent of these factors are poor levels of social support, worse symptoms of schizophrenia, and lower educational attainment by the caregiver. These findings highlight the need to provide community-based services and formal comprehensive supportive framework to cater for the needs of patients with schizophrenia and their caregivers. Further studies are needed to confirm these findings and to develop interventions targeted at alleviating the burden on caregivers of patients with schizophrenia in Nigeria. 

## Figures and Tables

**Table 1:** Sociodemographic characteristics of the participants *N* = 181.

Variables	Frequency	(%)
Caregiver Characteristics:		
Age range, mean (sd): 44.8 (±8.3)		
Gender		
Male	72	(39.8)
Female	109	(60.2)
Employment status		
Employed	101	(55.8)
Unemployed	80	(44.2)
Educational level		
At least secondary	107	(59.4)
Less than secondary	74	(40.6)
Relationship with patient		
Parent	81	(44.8)
Siblings	60	(33.2)
Spouse	9	(4.9)
Children	31	(17.1)
Patient characteristics:		
Gender		
Male	86	(47.5)
Female	95	(52.5)
Employment status		
Employed	54	(29.8)
Unemployed	127	(70.2)
Family Size, mean (sd): 6.19 (±1.35)		

**Table 2:** Clinical characteristics of the patients.

Variable	Mean (SD)
Positive PANSS scale score	21.87 (6.54)
Negative PANSS scale score	13.33 (3.24)
General PANSS scale score	35.63 (9.42)
Total PANSS score	70.83 (16.78)
Duration of illness (years)	34.63 (6.64)
Number of episodes	5.50 (2.31)
Number of admission	1.10 (1.12)

**Table 3:** Profile of Zarit burden interview (ZBI) scores.

Variable	Mean (SD)
Total ZBI scores	40.98 (16.7)
“Financial/physical strain” domain score	16.72 (4.7)
“Time/dependence strain” domain score	12.14 (3.9)
“Emotional strain” domain score	5.47 (2.4)
“Uncertainty” domain score	4.15 (2.1)
“Self-criticism” domain score	5.18 (2.1)

**Table 4:** Correlates of caregivers burden on univariate analysis.

Variable	r*	*P*
Dependent variable: “financial/physical strain”		
Duration of illness	0.197	0.008
Number of people in household	−0.167	0.025
Total PANSS scores	0.609	<0.001

Dependent variable: “time/dependence strain”		
Duration of illness	0.188	0.011
Number of people in household	−0.174	0.019
Total PANSS scores	0.684	<0.002
Number of episodes	0.157	0.032

Dependent variable: “emotional strain”		
Total PANSS scores	0.747	<0.001

Dependent variable: “uncertainty”		
Age of caregiver	0.304	0.002
Duration of illness	0.763	<0.001

Dependent variable: “self-criticism”		
Hours of patient-caregiver contact (weekly)	−0.547	<0.001
Total PANSS scores	0.314	0.012

KEY: *r*: Pearson's correlation coefficient.

**Table 5:** Multivariate analysis of the correlates of caregiver burden.

	Unstandardized coefficient		Standardized coefficient		*P*	95% C.I	*R* ^2^
	*B *	S.E	Beta	*t*			
Dependent variable: “financial/physical strain” domain score							
Poor social support	3.533	0.690	0.386	5.122	<0.001	2.167–4.899	25.3
Below secondary education	1.499	0.411	0.265	3.645	<0.001	1.685–2.313	17.1
Total PANSS	0.052	0.017	0.249	3.094	0.002	0.019–0.086	12.1

Dependent variable: “time/dependence strain” domain score							
Total PANSS	0.057	0.017	0.278	3.333	0.001	0.023–0.091	14.1
Poor social support	2.791	0.705	0.309	3.960	<0.001	1.395–4.186	24.9
Below secondary education	1.581	0.420	0.283	3.765	<0.001	0.750–2.413	22.0

Dependent variable: “emotional strain” domain score							
Total PANSS	0.054	0.007	0.461	7.246	<0.001	0.039–0.068	27.4
Poor social support	1.856	0.307	0.364	6.051	<0.001	1.249–2.463	25.8
Below secondary education	0.556	0.181	0.176	3.073	0.003	0.198–0.915	11.3
Female caregiver	0.914	0.312	0.210	2.933	0.04	0.297–1.531	3.4

Dependent variable: “Uncertainty” domain score							
Duration of illness	0.167	0.042	0.291	3.947	<0.001	0.083–0.250	23.7

Dependent variable: “Self-criticism” domain score							
Hours of contact	−0.195	0.032	−0.481	−6.175	<0.001	0.160–0.132	29.9

KEY: *R* ^2^: Percentage variance.

**Table 6:** Exploratory factor analysis of ZBI items with varimax rotation.

Item	“Financial/physical strain”	“Time/dependency strain”	“Emotional strain”	“Uncertainty”	“Self-criticism”
15	0.753	—	—	—	—
11	0.697	—	—	—	—
17	0.693	—	—	—	—
10	0.650	—	—	—	—
12	0.586	—	—	—	—
1	—	0.776	—	—	—
6	—	0.713	—	—	—
2	—	0.711	—	—	—
4	—	0.546	—	—	—
9	—	—	0.905	—	—
3	—	—	0.827	—	—
7	—	—	—	0.767	—
19	—	—	—	0.687	—
21	—	—	—	—	0.889
22	—	—	—	—	0.812
Eigen value	6.56	1.97	1.45	1.34	1.14
Exp. var (%)	31.2	9.4	6.9	6.4	5.4

KEY: Exp var (%): Explained variance (%). ZBI: Zarit burden interview.
